# Illness progression in chronic fatigue syndrome: a shifting immune baseline

**DOI:** 10.1186/s12865-016-0142-3

**Published:** 2016-03-10

**Authors:** Lindsey Russell, Gordon Broderick, Renee Taylor, Henrique Fernandes, Jeanna Harvey, Zachary Barnes, AnneLiese Smylie, Fanny Collado, Elizabeth G. Balbin, Ben Z. Katz, Nancy G. Klimas, Mary Ann Fletcher

**Affiliations:** Department of Medicine, University of Alberta, Edmonton, AB Canada; Department of Occupational Therapy, University of Illinois at Chicago, Chicago, IL USA; Department of Medicine, University of Miami, Miami, FL USA; Miami Veterans Affairs Medical Center, Miami, FL USA; Institute for Neuro-immune Medicine, Nova Southeastern University, Suite 3440 University Park Plaza, 3424 South University Drive, Fort Lauderdale, FL 33328 USA; Division of Infectious Diseases, Ann & Robert H Lurie Children’s Hospital of Chicago, Chicago, IL USA

**Keywords:** Cytokines, Chronic fatigue, Menopause and immunity, Immune signaling, Classification model

## Abstract

**Background:**

Validation of biomarkers for myalgic encephalomyelitis/chronic fatigue syndrome (ME/CFS) across data sets has proven disappointing. As immune signature may be affected by many factors, our objective was to explore the shift in discriminatory cytokines across ME/CFS subjects separated by duration of illness.

**Methods:**

Cytokine expression collected at rest across multiple studies for female ME/CFS subjects (i) 18 years or younger, ill for 2 years or less (*n* = 18), (ii) 18–50 years of age, ill for 7 years (*n* = 22), and (iii) age 50 years or older (*n* = 28), ill for 11 years on average. Control subjects were matched for age and body mass index (BMI). Data describing the levels of 16 cytokines using a chemiluminescent assay was used to support the identification of separate linear classification models for each subgroup. In order to isolate the effects of duration of illness alone, cytokines that changed significantly with age in the healthy control subjects were excluded a priori.

**Results:**

Optimal selection of cytokines in each group resulted in subsets of IL-1α, 6, 8, 15 and TNFα. Common to any 2 of 3 groups were IL-1α, 6 and 8. Setting these 3 markers as a triple screen and adjusting their contribution according to illness duration sub-groups produced ME/CFS classification accuracies of 75–88 %. The contribution of IL-1α, higher in recently ill adolescent ME/CFS subjects was progressively less important with duration. While high levels of IL-8 screened positive for ME/CFS in the recently afflicted, the opposite was true for subjects ill for more than 2 years. Similarly, while low levels of IL-6 suggested early ME/CFS, the reverse was true in subjects over 18 years of age ill for more than 2 years.

**Conclusions:**

These preliminary results suggest that IL-1α, 6 and 8 adjusted for illness duration may serve as robust biomarkers, independent of age, in screening for ME/CFS.

**Electronic supplementary material:**

The online version of this article (doi:10.1186/s12865-016-0142-3) contains supplementary material, which is available to authorized users.

## Background

Myalgic encephalomyelitis/chronic fatigue syndrome (ME/CFS) is a complex and poorly understood illness [1] that affects up to 4 million individuals [2, 3] and costs an estimated $35 billion per year in lost productivity and health care [4, 5] in the US alone. It presents in a significant fraction of cases as a sequelum of acute infection [6, 7] and while men are susceptible, ME/CFS affects a disproportionate number of women [8] in a ratio of up to 6:1 [9]. Moreover recent preliminary work by our group suggests that this illness may present different patterns of cytokine expression in women as it does in men [10]. Biomarkers for ME/CFS have been reported [11] but validation in external datasets has proven difficult [12, 13] at least in part because of the heterogeneous composition of subject cohorts, in particular with regard to sex and age and the confounded effects thereof. Indeed in recent work, Lewis et al. [14] conclude that older ME/CFS patients (age >50 years) demonstrate a disease phenotype very different from that of younger patients (16–29 years) who may be more likely to develop CFS following an infection, including greater autonomic dysfunction and depressive symptoms. In addition Hornig et al. [15] demonstrated cytokine profiling differences between short and long duration of illness of age matched cohorts of ME/CFS compared to healthy controls. Menopausal status also emerged a segregating feature in differentiating sub-classes of ME/CFS in work by Vollmer-Conna et al. [16].

Influences of sex on immune function have long been recognized. The higher risk of women developing autoimmune diseases suggests that the latter may be mediated by sex steroids [17]. Accordingly, both cellular and humoral immune responses may be modulated during the different phases of the menstrual cycle with NK cell cytolytic activity decreasing in the pre-ovulatory period [18, 19] and a shift towards Th-2 immunity occurring in the luteal phase [20]. Our work has shown that decreased NK cell cytotoxic function is a consistent feature in a group of older ME/CFS subjects [21]. In addition, Cannon et al. [22] reported that while neutrophil count normally increased from the follicular to luteal phase in healthy women, it persisted at luteal levels in ME/CFS.

Other immunological features have also been reported. For example, postmenopausal women typically show a reduction in total lymphocyte count with this decrease involving mainly B and CD4+ T lymphocyte subpopulations [23]. These same authors report that women with premature menopause had lower CD4+, higher CD8+ and higher NK cell counts than fertile women of the same age. More recent work has confirmed that estrogen alters levels of cortisol, the major mediator of the HPA axis and immune responses, during menstrual cycles and in post-menopausal women [24, 25]. These observations argue in favor of a stratification of subjects on the basis of menopausal status when studying illnesses affecting immune and endocrine function. In addition there are changes in the risk for autoimmune and chronic inflammatory diseases between both pre and post-menopausal women compared to men [26]. Aging is another factor to take into account as it is associated with an increased prevalence of chronic inflammatory diseases and immune dysregulation [27, 28]. Progression of illness-specific changes in immune function are therefore confounded with changes expected as a result of normal ageing and menopause. In this study we attempted to identify shifts in the expression of 16 cytokines that might be primarily driven by illness while controlling for those that are associated with normal immune ageing and menopause. In an overall group of *n* = 68 female ME/CFS subjects and *n* = 73 healthy control subjects, ranging in age from 12 to 75 years, we found that the expression of IL-1α, 6, and 8 in blood supported a classification accuracy of 75–88 % for ME/CFS when adjusted for duration of illness.

## Methods

### Subject cohorts

The cohort of young subjects <18 years of age is described in previously [7]. In brief, this cohort consisted of *n* = 12 adolescent subjects with post-infectious ME/CFS resulting from infectious mononucleosis (IM) (mean age 16 years, mean BMI 24) and case matched recovered control subjects (mean age 16 years, mean BMI 23) recruited and assessed as part of a 2-year prospective study 301 adolescents recruited from the greater Chicago area. Adolescents were identified via school nurses (middle school, high school and college/university), pediatric practices, including the Pediatric Practice Research Group and the Virology Laboratory of Children’s Memorial Hospital (now the Ann & Robert H Lurie Children’s Hospital of Chicago). Clinical evaluation included laboratory tests to rule out medical causes of ME/CFS (e.g., chemistry panel, complete blood count, erythrocyte sedimentation rate, liver chemistries, urine toxicology, urinalysis and thyroid function tests). The examining physician made a diagnosis of ME/CFS, ME/CFS-explained, or recovered on each subject. These diagnoses were then blindly reviewed by an expert panel using the Jason revision for pediatrics [29] of the Fukuda criteria [1]. Each subject also completed the Chalder Fatigue Scale at 6, 12 and 24 months [30], along with the Youth Medical Questionnaire, Modifiable Activity Questionnaire, Sleep Assessment Questionnaire, and the Structured Clinical Interview for the DSM-IV Child Version, Child/Young Adult Behavior Checklist, Child/Young Adult Self-Report, and Life Events and Difficulty Questionnaire [31]. At 24-months post-IM onset, 13 subjects satisfied the criteria for ME/CFS, all of whom were young women. Plasma samples were available for *n* = 1 and *n* = 9 of these 13 ME/CFS patients at 12 and 24-months after diagnosis with IM respectively. These were case matched with samples at 24 months from *n* = 12 recovered controls based on age (+/− 1 year) and Tanner stage (4 or 5).

The second cohort consisted of adult female ME/CFS patients (*n* = 40; mean age 50, mean BMI 27) that were recruited from the ME/CFS and Related Disorders Clinic at the University of Miami. A diagnosis of ME/CFS was made using the International Case Definition [1, 31]. Healthy female controls (*n* = 59; mean age 53, mean BMI 26) were recruited from the same metropolitan area under a NIH funded study. This cohort was enriched by integrating a third smaller subject group consisting of *n* = 10 female ME/CFS subjects (mean age 43 years, mean BMI 27) and 10 female healthy control (HC) subjects (mean age 45 years, mean BMI 32) recruited as part of a separate ongoing study and assessed according to the same protocols and case definition criteria.

This overall set of female ME/CFS subjects recruited across the 3 studies was combined and then separated into 3 groups according to age and illness duration as follows: (i) adolescent early course subjects, 18 years or younger, ill for 2 years or less (*n* = 18), (ii) adult pre-menopausal mid-course subjects, 18–50 years of age, ill on average for 7 years (*n* = 22), and (iii) adult peri/post-menopausal late course subjects, age 50 years or older (*n* = 28), ill for 11 years on average. Control subjects were matched for age and body mass index (BMI).

All ME/CFS study subjects had a SF-36 summary physical score (PCS) below the 50th percentile, reflecting a level of impairment exceeding the population norm. Exclusion criteria for ME/CFS included all of those listed in the current Centers for Disease Control (CDC) ME/CFS case definition, including the listed psychiatric exclusions, as clarified in the International CFS Working Group [32]. All ME/CFS subjects were assessed for psychiatric diagnosis at the time of recruitment with the Composite International Diagnostic Instrument [29]. Based on this assessment, we excluded subjects with DSM IV diagnoses for psychotic or melancholic depression, panic attacks, substance dependency, or psychoses as well as any subjects currently suicidal. We also excluded subjects with Borderline or Antisocial Personality Disorder. Subjects had no history of heart disease, COPD, malignancy, or other systemic disorders that would be exclusionary, as clarified by Reeves et al. [32].

#### Ethics statement

Informed consent to participate in the study was obtained for all studies, including the Chicago-based prospective study, from all participants (or their parent or guardian in the case of children under 16). Consent and study protocols were approved as appropriate by the Institutional Review Board of the Department of Veterans Affairs Medical Research Development Committee (protocol #4987.76) and the Children’s Memorial Hospital (now Ann & Robert H Lurie Children’s Hospital of Chicago) (IRB#2009-13726). Ethics review and approval for data analysis was also obtained by the IRB of the University of Alberta Health Research Ethics Board-Biomedical Panel (protocols Pro00018859 and Pro00004286).

### Cytokine profiles

In all cohorts morning fasting AM blood samples were collected into ethylene diamine tetra acetic acid (EDTA) anticoagulant tubes. Plasma was separated within 2 h of collection and stored at −80 °C until assayed. We measured 16 cytokines in plasma using Quansys reagents and instrument (Quansys Biosciences, Logan, Utah). These were interleukin (IL) 1α, 1β, 2, 4, 5, 6, 8, 10, 12p70, 13, 15, 17 and 23 as well as interferon gamma (IFNγ), tumour necrosis factor alpha (TNF α) and lymphotoxin-α (LTα) The Quansys Imager, driven by an 8.4 megapixel Canon 20D digital SLR camera, supports 96 well plate based chemiluminescent imaging. The Q-Plex™ Human Cytokine-Screen (16-plex) is a quantitative enzyme-linked immunoabsorbent assay (ELISA)-based test where sixteen distinct capture antibodies have been absorbed to each well of a 96-well plate in a defined array. The range of the cytokine concentrations used in the standard calibration samples were adjusted for each cytokine along with sample exposure time to provide the most reliable comparison possible between ME/CFS patients and controls across the range of cytokine concentrations known and expected in plasma. For the standard curves, we used the second order (*k* = 2) polynomial regression model (parabolic curve): Y_p_ = b_0_ + b_1_X^1^.... + b_k_X^k^, where Y_p_ is the predicted outcome value for the polynomial model with regression coefficients b_1_ to b_k_ for each degree and y intercept b_0_. Details of the protocol and assay variability have been reported previously by our group [33–35]. In brief, replicate error in the measurement of IL-1α, IL-10 and IL-17 using this panel does not readily support subject-to-subject comparisons however the resolution is more than adequate for use in comparisons across patient groups as a whole. Values below detection limit were replaced with the lowest concentration recorded for each specific cytokine within each subject group (see Supplemental Table S3 in [34]). Statistics describing the distribution of values obtained in each subject subgroup are shown in Additional file 1: Table S1(a) and (b). To verify consistency in the collection and handling of blood samples across the Chicago and Miami clinical sites we performed a principal component analysis (PCA) [36] capturing co-expression patterns of the 16 cytokines in healthy control subjects. Additional file 2: Figure S1 shows the Hotelling T^2^ statistic [37], a measure of departure from the co-expression model, for each of the samples collected in Chicago from the adolescent control subjects and those collected in Miami from the two groups of adult subjects. Results confirmed that the middle-aged and adolescent groups were statistically comparable in broad co-expression despite samples being collected at two separate sites. In addition the deviation cytokine co-expression in the healthy subjects aged 50 years and older aligns with observations of immune shift in healthy women of post-menopausal age.

The full set of de-identified, coded and normalized data used in this work is available in Additional file 3: Table S6. This same file contains symptom severity scores based on the Multidimensional Fatigue Inventory (MFI) [38] for all ME/CFS subjects older than 18 years of age (Additional file 3: Table S7). Symptom severity in the adolescent cohort (age ≤18 years) was assessed using the Chalder Fatigue Scale [30] and is described separately in Katz et al. [7].

### Statistical analysis

All cytokine concentrations were log base 2 transformed (log2), then centered and range adjusted by way of s standard z-score normalization. In assessing the significance of changes across multiple groups the parametric analysis of variance (ANOVA) was applied as was the non-parametric Kruskall-Wallis test. Null hypothesis *p*-values obtained through these tests were used as criteria for selecting those cytokines that did not vary significantly between age groups in the healthy control subjects only. The significance of pair-wise differences in concentration across any two groups was estimated using a standard parametric two-tailed *t*-test in conjunction with the non-parametric Wilcoxon ranksum test.

Linear discriminant classification models were generated that assigned subjects to ME/CFS or control groups based on expression of a subset of the 16 cytokines measured. Selection of specific cytokines for inclusion into the model was conducted using a step-wise selection procedure whereby candidates are individually added and removed from the discriminant function based on their partial F statistic [39]. According to this model an observed row *x* from the sample array is classified into group I rather than group J if 0 < B_0_ + x*B, where the coefficient vector B and intercept vector B_0_ are estimated from the data. Classifier performance was assessed in terms of overall accuracy (correct rate), sensitivity, specificity, positive predictive value (PPV), and negative predictive value (NPV). The evolution of linear discriminant coefficients with duration of illness was expressed as a second order polynomial regression model (parabolic curve): Y_p_ = b_0_ + b_1_X.... + b_k_*X*^2^, where Y_p_ is the predicted outcome value for the polynomial model with regression coefficients b_1_ to b_k_ for each degree and intercept b_0_. These calculations were performed using the *classify* and *classperf* functions available in the MatLab Statistics Toolbox and the MatLab Bioinformatics Toolbox (The MathWorks, Inc., Natick, MA).

Correlation of MFI symptom severity with cytokine expression reported in Additional file 1: Table S4, was based on the partial Spearman rank correlation corrected for BMI and duration of illness. This measure and its application are described further in Emmert-Streib [40] as well as Magwene and Kim [41]. Partial correlation was calculated using the *partialcorr* function available in the MatLab Statistics Toolbox (The MathWorks, Inc., Natick, MA).

## Results

### Differences in cytokine expression between healthy and ME/CFS populations

Differences in the expression of individual markers between HC subjects and subjects diagnosed with ME/CFS) were assessed in each of the age and duration of illness group using the non-parametric ranksum test and the standard two-tailed *t* test applied to the log2-transformed cytokine data (Additional file 1: Table S2). Significant differences in both the mean (*t* test) and median log-transformed expression values (ranksum test) were observed between the HC and ME/CFS (*p* < 0.05) in at least one of the 3 subgroups for 7 out of the 16 cytokines measured. These differences were especially abundant in the sub-group of subjects aged 50 years or more and ill for 11 years on average. In the latter, IL-4, IL-5, IL-12 and LTα were increased in expression in ME/CFS, while IL-8 and IL-15 were expressed at lower levels in ME/CFS. Conversely increased expression values for IL-8 were observed in adolescent ME/CFS subjects with log-transformed concentrations of IL-23 being expressed at significantly lower levels in this sub-group.

### Validating a prior classification model

As cytokines are not expressed independently of one another [34], we had previously applied both a sequential step-wise selection procedure and an all-possible subsets procedure to identify subsets of cytokines that when used as the basis of a linear classification model might provide a co-expression signature characteristic of ME/CFS in the adolescent cohort used here [35]. Use of the sequential selection procedure identified IL-1a, IL-6, IL-8, IL-13 and IL-23 as potential markers of ME/CFS in this adolescent population (Table 1). Of these cytokines, IL-6, IL-8 and IL-23 were also selected by the all-possible subsets method forming the basis for a reduced consensus model. As described in Broderick et al. [35] random sub-sampling of the adolescent subjects indicated that IL-6 and 8 provided an especially robust basis for a minimal classification model of post-infectious ME/CFS in our adolescent population. Even this minimal model of ME/CFS supported a classification accuracy of close to 80 % in the adolescent training set. However as shown in Table 1, all three variants of this classification model extrapolated poorly to the older mid-course and late course subject sub-groups with accuracies of less than 50 %.

**Table 1:** Extrapolation of classification models identified in adolescent CFS (Broderick et al., 2012) to adult pre and post-menopausal groups

Classification based on	Stepwise model of IL-1α, 6,8,13,23	Consensus model of IL-6, 8, and 23	Minimal model of IL-6 and 8
(a) Training set: Age < =18 years			
Correct rate	0.93	0.86	0.79
Error rate	0.07	0.14	0.21
Sensitivity	0.94	0.89	0.78
Specificity	0.92	0.83	0.79
Positive predictive value	0.89	0.80	0.74
Negative predictive value	0.96	0.91	0.83
(b) Test set: 18 < Age < = 50 years			
Correct rate	0.49	0.26	0.35
Error rate	0.51	0.74	0.65
Sensitivity	0.09	0.23	0.09
Specificity	0.90	0.29	0.62
Positive predictive value	0.50	0.25	0.20
Negative predictive value	0.49	0.26	0.39
(c) Test set: Age > 50 years ^a^			
Correct rate	0.46	0.29	0.39
Error rate	0.54	0.71	0.61
Sensitivity	0.00	0.32	0.14
Specificity	0.93	0.25	0.64
Positive predictive value	0.00	0.30	0.29
Negative predictive value	0.48	0.27	0.43

^a^ *n* = 28 of 47 HC to match ME/CFS age and BMI

### Selecting cytokines broadly conserved across age and BMI

Even in healthy individuals cytokine expression is influenced by a variety of factors such as age and BMI [17, 42–44]. Indeed when examining changes in cytokine expression in healthy individuals alone we found that the majority of the 16 cytokines measured here changed significantly in expression (Table 2a). Despite transformations to the data, significance analysis based on classical ANOVA was further verified using the Kruskall-Wallis non-parametric test and variable selection was based on the more conservative result. Only IL-6, TNF-α and LTα levels were not significantly different among healthy control subjects across all 3 age subgroups (Table 2a). Expression of IL-1α, 8 and 15 was statistically stable only across subgroups composed of predominantly premenopausal healthy individuals (Table 2b). While significant differences in BMI were seen in healthy subjects between the adolescent subgroup and the subgroups with older subjects, the overall range of values was such that the vast majority of subjects were non-obese (58 of 73 with BMI <30 kg/m^2^) [45].

**Table 2:** Results of one-way ANOVA and Kruskal–Wallis tests in healthy control (HC) subjects only (a) across all 3 age groups, and (b) across the 2 age groups with predominantly premenopausal subjects (age ≤50 years)

(a) HC all age groups			(b) HC groups age ≤50 years only	
	p ANOVA	p Kruskalwallis	p ANOVA	p Kruskalwallis
BMI	0.00	0.01	0.00	0.00
IL-1α	*0.23*	0.04	*0.12*	*0.12*
IL-1β	0.00	0.00	0.00	0.00
IL-2	*0.27*	0.01	*0.32*	0.02
IL-4	0.00	0.00	*0.18*	0.01
IL-5	0.00	0.00	0.00	0.00
IL-6	*0.99*	*0.89*	*0.91*	*0.95*
IL-8	*0.08*	0.01	*0.27*	*0.06*
IL-10	0.00	0.00	0.00	0.00
IL-12p70	0.00	0.00	0.02	0.01
IL-13	0.00	0.00	0.00	0.00
IL-15	*0.22*	0.04	*0.98*	*0.41*
IL-17	0.00	0.00	0.00	0.01
IL-23	0.00	0.00	0.00	0.00
IFNγ	0.02	0.02	0.00	0.01
TNFα	*0.89*	*0.33*	*0.82*	*0.17*
LTα	*0.11*	*0.75*	*0.09*	*0.67*

*p* = > 0.10

In an attempt to remove the confounding effects of age and BMI on cytokine expression, we constructed a new set of classification models using only IL-1a, IL-6, IL-8, IL-15, TNF-α and LTα as candidate markers since these were reasonably invariant in healthy subjects. Cytokines IL-1α, TNF-α and LTα, IL-6 and IL-8 had been previously selected as discriminatory markers [35] in the adolescent set and were retained here as the basic model (Table 3) producing a classification accuracy of 88 % in this subgroup. Repeating the stepwise selection procedure for the middle aged mid-course subgroup led to the identification of IL-1α and IL-15 as being the best markers for this subgroup yielding an accuracy of 72 %. Likewise in the predominantly post-menopausal late-course group, IL-6, IL-8, IL-15 and TNF-α were selected to deliver a classification accuracy of 84 %. As these subgroup specific marker sets overlap, the applicability of the simple model identified in the adolescent subgroup was tested on both other age and illness duration groups yielding accuracies of 37 and 48 %. To explore whether this decrease in performance was related to the choice of cytokines, we constrained the structure of the classification model to be based on IL-1α, IL-6 and IL-8 but allowed the coefficients to be tuned for each of the illness subgroups. When coefficients were tuned in this way the classification accuracy based on IL-1α, IL-6 and IL-8 rose to 77 and 75 % in the middle-aged and post-menopausal subgroups respectively (Table 3). As IL-1α, IL-6 and IL-8 were selected from a candidate set of cytokines that were reasonably invariant across age and BMI in healthy subjects this result suggests that duration of illness may be a main factor driving the need for parameter tuning across ME/CFS subgroups, at least in the group of cytokines measured here. This choice of markers would also be consistent with a cursory analysis of illness severity showing that IL-1α and IL-8 in particular display a correlation of at least marginal significance (*p* ≤ 0.07) or better with the general fatigue, physical fatigue and reduced activity components of the MFI (Additional file 1: Table S4).

**Table 3:** Best Stepwise models selected for each set from cytokines stable across HC groups of age ≤50 years

Subject sub-group	Classification model		
	Training selection	Training selection	
(a) Training set: Age < =18 years	IL-1α, 6, 8	IL-1α, 6, 8	
Correct rate	0.88	0.88	
Error rate	0.12	0.12	
Sensitivity	0.89	0.89	
Specificity	0.88	0.88	
Positive predictive value	0.84	0.84	
Negative predictive value	0.91	0.91	
	Training selection	Test set	Parameters tuned
(b) Training set: 18 < Age < = 50 years	IL-1α, 15	IL-1α, 6, 8	IL-1α, 6, 8
Correct rate	0.72	0.37	0.77
Error rate	0.28	0.63	0.23
Sensitivity	0.73	0.09	0.77
Specificity	0.71	0.67	0.76
Positive predictive value	0.73	0.22	0.77
Negative predictive value	0.71	0.41	0.76
(c) Training set: Age > 50 years ^a^	IL-6, 8, 15, TNFα	IL-1α, 6, 8	IL-1α, 6, 8
Correct rate	0.84	0.48	0.75
Error rate	0.16	0.52	0.25
Sensitivity	0.86	0.18	0.75
Specificity	0.82	0.79	0.75
Positive predictive value	0.83	0.45	0.75
Negative predictive value	0.85	0.49	0.75

^a^ *n* = 28 of 47 HC to match CFS/ME age and BMI

### A classification model corrected for duration of illness

The classification results above suggest that IL-1α, IL-6 and IL-8 may be broadly applicable as ME/CFS illness markers but that their contribution should be adjusted based on duration of illness and perhaps other related covariate factors. To assess the variability of such adjustments the coefficients for these cytokines in the linear classification model were estimated repeatedly on 50 random subsets of 10 healthy control and 10 ME/CFS subjects in each illness duration subgroup. Subjects would be assigned to the ME/CFS class if 0 < α_0_ + α_1_× [IL-1a] + α_2_ × [IL-6] + α_3_ × [IL-8], where [x] is the z score normalized concentration of cytokine x based on the mean and standard deviation values listed in Additional file 1: Table S3. Results of this piece-wise optimal tuning of classification coefficients are shown in Table 4 supporting an accuracy in classification of approximately 75 ± 8 % standard error on these smaller random subsets. Sensitivity values were 75 ± 12 % to 78 ± 9 % in the adult subsets. Corresponding specificity levels exceeding 73 ± 11 % ranging up to 83 ± 8 %.

**Table 4:** Performance statistics for classification models built from 50 random subsets of 10 healthy and 10 CFS/ME subjects

Classification based on IL-1α, 6 and 8	Median (MADM Std Err ^a^)	Mean (Std Err)
(a) 50 random subsets: Age < =18 years		
Correct rate	0.90 (0.01)	0.89 (0.06)
Sensitivity	0.90 (0.00)	0.92 (0.06)
Specificity	0.85 (0.01)	0.86 (0.08)
Positive predictive value	0.87 (0.01)	0.87 (0.07)
Negative predictive value	0.90 (0.00)	0.92 (0.06)
(b) 50 random subsets: 18 < Age < = 50 years		
Correct rate	0.75 (0.01)	0.76 (0.08)
Sensitivity	0.80 (0.02)	0.78 (0.09)
Specificity	0.70 (0.02)	0.73 (0.11)
Positive predictive value	0.73 (0.01)	0.75 (0.10)
Negative predictive value	0.78 (0.01)	0.77 (0.08)
(c) 50 random subsets: Age > 50 years ^b^		
Correct rate	0.80 (0.01)	0.79 (0.08)
Sensitivity	0.80 (0.02)	0.75 (0.12)
Specificity	0.80 (0.01)	0.83 (0.08)
Positive predictive value	0.80 (0.01)	0.82 (0.08)
Negative predictive value	0.80 (0.02)	0.77 (0.09)

^a^MADM Std Err = 1.4826 × MADM/*√n*

^b^ *n* = 28 of 47 HC to match ME/CFS age and BMI

**Fig. 1:**
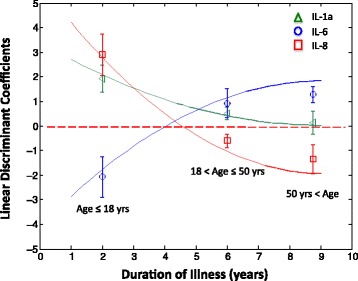
Relative contribution to classification of ME/CFS subjects versus healthy control subjects across duration of illness for cytokines largely unaffected by age and BMI. Average value with standard error for the coefficients of IL-1α, IL-6 and IL-8 in a linear model for classification of ME/CFS subjects as estimated across 50 random subsets of *n* = 10 ME/CFS and *n* = 10 healthy control subjects sampled from subgroups of differing illness duration

The corresponding mean and median values for these optimally tuned coefficients are listed in Table 5 and shown in Fig. 1. These indicate that duration of illness influences both the magnitude and the polarity of the contribution made by each cytokine in determining membership to the ME/CFS class. The coefficient α_1_ for IL-1α suggests that increased levels of the latter are most characteristic of ME/CFS in the early course of illness but that this feature decreases in importance as illness progresses. Coefficients α_2_ and α_3_ actually reverse in polarity as illness progresses with a combination of lower than average IL-6 and higher than average IL-8 levels being more discriminatory for early stage ME/CFS but the reverse pattern being more prominent in subjects with more established illness. Changes in the intercept α_0_ and the coefficients α_1_, α_2_, α_3_ with respect to duration of illness were captured using a simple second order polynomial of the form α_i_ = β_0_ + β_1_ × (years ill) + β_2_ × (years ill)^2^. This regression model is presented in Table 6. Results show that in the case of all classification coefficients, the duration of illness is a highly significant contributor (*F* > 32; *p* < 0.01). Indeed close to 60 % of the total variability in the classification coefficients for IL-6 and IL-8 are captured by duration of illness alone (R^2^ = 0.57). However only slightly more that 30 % of the total variability in the classification coefficients for IL-1α as well as that for the intercept are supported by changes in duration of illness. To evaluate the impact of this unexplained variability on classification accuracy we applied the simple protocol proposed in Additional file 4: Figure S2 where duration of illness alone is first used to calculate the appropriate values of the classification weights α_i_ for IL-1α, IL-6 and IL-8 using the β_i_ values listed in Table 6. Using the resulting estimates of coefficients α_0_, α_1_, α_2_, α_3_ in the linear classification model each subject was assigned a predicted ME/CFS (score >0) or non-ME/CFS status (score ≤0). The protocol based on duration of illness alone supported a classification accuracy of 63 % across the full range of illness duration, a performance comparable to that obtained with an optimal tuning based on all subjects (Additional file 1: Table S5). However important gaps in performance emerged across the different phases of illness. This was especially obvious for classification performed in the mid-range of illness duration (i.e. 6–7 years ill). These results illustrate that although duration of illness may be a highly significant contributor to the evolution of the classification coefficients (*p* < 0.01), other covariate factors related to illness progression may also play an important role, in particular during the transition from early to late phase.

**Table 5:** Distribution statistics for coefficients in linear discriminant model based on 50 random subsets of 10 healthy and 10 CFS/ME subjects in each age group

(a) Median (MADM-based Std Err ^a^)		Age < =18 years	18 < Age < = 50 years	Age > 50 years
Discriminant coefficients (*n* = 50)				
IL-1α	α_1_	1.93 (0.11)	0.51 (0.06)	0.14 (0.10)
IL-6	α_2_	−2.08 (0.17)	0.91 (0.12)	1.28 (0.07)
IL-8	α_3_	2.89 (0.18)	−0.60 (0.06)	−1.36 (0.12)
Constant term	α_0_	−1.86 (0.09)	−0.39 (0.05)	−0.49 (0.05)
Duration of illness (years)		2.00 (0.00)	6.50 (1.74)	8.50 (1.26)
(b) Mean (Std Err)				
Discriminant coefficients (*n* = 50)				
IL-1α	α_1_	2.41 (0.23)	0.53 (0.10)	0.14 (0.13)
IL-6	α_2_	−2.36 (0.19)	1.09 (0.13)	1.51 (0.13)
IL-8	α_3_	3.53 (0.27)	−0.92 (0.14)	−1.52 (0.19)
Constant term	α_0_	−2.07 (0.15)	−0.48 (0.06)	−0.61 (0.09)
Duration of illness (years)		2.00 (0.00)	7.09 (1.28)	10.64 (1.46)

^a^MADM Std Err = 1.4826 × MADM/*√n*

**Table 6:** Regression models for discriminant coefficients as a function of duration of illness

Regression Model		Intercept β_0_	β_1_ (years ill)	β_2_ (years ill ^2^)	R^2^	F	p
IL-1a coefficient	α_1_	3.42 (2.80, 4.04)	−0.75 (−0.97, −0.52)	0.04 (0.02, 0.06)	0.347	38.980	0.000
IL-6 coefficient	α_2_	−4.11 (−4.80, −3.43)	1.31 (1.06, 1.56)	−0.07 (−0.09, −0.05)	0.573	98.510	0.000
IL-8 coefficient	α_3_	5.84 (4.95, 6.73)	−1.71 (−2.04, −1.38)	0.09 (0.07, 0.12)	0.573	98.504	0.000
Constant term	α_0_	−2.56 (−3.00, −2.12)	0.45 (0.29, 0.62)	−0.02 (−0.04, −0.01)	0.310	32.986	0.000

() indicates the 95 % confidence interval

R^2^ is Pearson correlation coefficient and p is the null probability of the F statistic

## Discussion

There is mounting evidence suggesting that ME/CFS may be characterized by a significant imbalance in immune and endocrine function and that this imbalance may be perpetuated by an altered homeostatic response. Cytokine profiles that are characteristic of this persistent imbalance in immune regulation function have been reported previously by our group as well as by others described [10, 15, 34, 35]. Broad use of cytokine expression patterns as markers of illness is not without its challenges as these can be difficult to measure and are affected by a variety of factors even in a healthy population such as age, sex, BMI, etc.… Indeed Craddock et al. [46] demonstrated mathematically that basic differences in immune and endocrine regulatory wiring in men versus women is such that chronic response to insult in each sex can be significantly different. The concurrent measurement of multiple cytokines can serve to provide a measure of internal validation however the changes occurring as a result of illness progression as well as the shift that normally occurs in a healthy control population must also be considered. This study is a continued exploration of how characteristic cytokine expression might be in ME/CFS and how such a signature could be used to reliably isolate ME/CFS subjects from their healthy counterparts regardless of age and duration of illness. The cytokine-based classification model initially reported in Broderick et al. [35], showed promise when applied to the adolescent ME/CFS cohort reinforcing the notion that cytokine profiling may be a useful tool in supporting a diagnosis of ME/CFS. However this early model did not extrapolate well to a larger cohort of adult ME/CFS subjects. In any classification system it is important that we control for factors affecting the expression of those same candidate markers in healthy individuals. In this work we attempted to normalize and remove changes in immune signaling that are driven by age [17, 42, 43] and BMI [44] in a healthy population. As a significant segment of the healthy subjects used here as a comparator group straddled the average age of menopause we reasoned that this may be one of the factors affecting the stability of biomarker discovery in the broader ME/CFS population. In a recent review by Gamiero, Romão and Castelo-Branco [43] evidence suggests that healthy post-menopausal women exhibit elevated levels of IL-6 and IL-18 accompanied by decreased levels of TNF-α and LTα compared to their middle-aged pre-menopausal counterparts. While the mean levels of TNF-α and LTα exhibited a decreasing trend in healthy women over 50 years of age, these trends did not achieve statistical significance in this work. Levels of IL-18 were not measured in the current study but levels of IL-6 were also statistically similar across age groups in healthy subjects studied here. It is important to note that contrary to the studies reviewed in Gamiero, Romão and Castelo-Branco [43] the subgroups used here were based solely on age and that the expression levels of sex hormones were unavailable to confirm menopausal status. Nonetheless we found significantly higher IL-6 expression in the older ME/CFS subgroup compared to the middle-aged ME/CFS group. Though these changes could not be associated directly with menopausal status here, this observation nonetheless aligns with recent work by Boneva et al. [47] where ME/CFS was associated with early hysterectomy and/or menopause, linking this condition to the reduction or depletion of endogenous female sex hormones and to the possibility that menopause-like changes in immune signature may constitute an inherent component of ME/CFS pathology. Such a link has been observed in other complex illnesses with immune involvement [48].

After controlling for age and BMI, we found that it might be possible to support the diagnosis of ME/CFS in a broad population by adjusting the relative importance of IL-1α, IL-6 and IL-8 expression to the duration of illness. In a recent study, Hornig et al. [15] compared plasma cytokine profiles in ME/CFS subjects ill for 1.7 years on average (std.dev. 0.8) (mean age 40.5 years; std. dev. 13.6 years) with those ill for 15.6 years on average (std. dev. 8.2) (mean age 50.2 years; std. dev. 11.4) as well as with age-matched control subjects. As in the current work, the latter found a decrease in IL-1α and IL-8 in the ME/CFS cohort with longer duration illness compared to the group with a more recent onset (*p* < 0.01, <0.0001 respectively). These investigators also reported significantly depressed IL-6 concentrations in subjects with more established illness (*p* < 0.05). A similar decrease was also observed here when comparing IL-6 levels in the late stage ME/CFS subgroup (ill for 11 years on average) with the early stage subgroup ill for 2 years or less. However we also found that this decrease in IL-6 expression was even more pronounced in the mid-course subgroup (average illness duration of 7 years) and that levels increased again in the late course but remained below those expressed in the early stage. In comparing these works further it is important to recall that Hornig et al. [15] used a arbitrary threshold of 3 years to define long versus short illness duration and that subject groups spanned across the average age of menopausal onset (approx. 50 years) [49, 50]. Despite these differences in granularity and subgroup definition the subjects we studied in this work appear to display cytokine profiles similar to those found in the larger ME/CFS population studied by Hornig et al., [15].

Extending beyond group-wise comparisons, our study attempts to identify a subset of cytokines that when used together might provide a highly discriminatory signature for ME/CFS that is robust to differences in age, BMI and could be adjusted for duration of illness. The panel proposed here based on the co-expression of IL-1α, IL-6 and IL-8 supports an optimal accuracy of 78–88 % in a random internal cross-validation. More importantly the relative contribution of each of these cytokines seems to shift with duration of illness. A co-expression pattern of increased IL-1α and IL-8 in the context of decreased IL-6 emerged as characteristic of early course illness compared to age and BMI-matched control subjects. In contrast elevated IL-1α, and IL-6 co-expressed in the context of lower than average IL-8 was a more abundant pattern in mid and late course ME/CFS subjects compared to age and BMI-matched controls. Once again important to note that Hornig et al. [15] focused on features that discriminate ME/CFS subjects with early-stage illness from those with established illness. In this work we focus on features that distinguish ME/CFS from age and BMI-matched healthy control subjects, examining instead how these might change at different stages of illness.

This remains an exploratory first analysis of how a set of blood-borne immune markers might be used to support the accurately diagnosis ME/CFS in an efficient and effective way. Several challenges persist in moving to larger multi-site validation studies. For example even though all assays were performed by the same diagnostic laboratory (Miami) and collected using the same prescribed protocol, the phlebotomy teams were specific to each site and future studies should strive to better capture such potential sources of bias. Nonetheless the current work describes a protocol that could be useful in assessing a much broader variety of potential markers such as neuropeptide Y (NPY), previously shown by our group to be a correlate of ME/CFS severity [51]. Extending the marker set to include candidates measured in other physiological compartments would also be useful. Studies of cerebral spinal fluid (CSF) from ME/CFS subjects have pointed to a characteristic decrease in IL-10 levels confirming illness effects reaching beyond the peripheral circulation to the central nervous system [52]. While the panel based on IL-1α, IL-6 and IL-8 levels in peripheral blood described here shows initial promise it remains paramount to further validate in a larger and even more varied cohort to ensure a robustness that would be compatible with clinical use. In keeping with this, it is important that these biomarkers remain reliable in the context of the often complex and broad set of co-morbid conditions afflicting ME/CFS sufferers.

## Conclusions

While the markers proposed here show promise, the present study highlights how progression of the illness itself may obscure the validation of potentially useful markers and proposes a regression approach that might be used to address this challenge. Though direct translation into clinical use may be premature at this time, we believe that insights such as these are important to further understanding the shifting immune biology of ME/CFS.

### Availability of supporting data

The data set supporting the results of this article is included within the article as Additional file 2.

## Additional files

Additional file 1: Tables S1, S2, S3. Supplemental tables containing summary statistics of the data collected in each cohort segment, group-wise comparative statistics and normalization constants for each cytokine species. **Table S4.** contains Spearman correlation and corresponding null probability values for pair-wise correlation of the Multidimensional Fatigue Inventory (MFI) with IL-1α, IL-6 and IL-8 levels in subjects older than 18 years of age. Table S5 contains the performance of classification based on IL-1α, IL-6 and IL-8 levels where the relative contribution of each cytokine is adjusted on the basis of duration of illness *only* as described in Additional file 4: Figure S2. (XLS 51 kb) Additional file 2: Figure S1. Site-to-site variability in samples from healthy control subjects. Hotelling’s T squared residual distance separating samples collected in all 3 groups of healthy control subjects from a principal component analysis (PCA) model describing the co-expression patterns linking 16 cytokines. Middle-aged and adolescent groups were statistically comparable (*p* = 0.839) despite samples being collected at separate sites. (PDF 37 kb) Additional file 3: Table S6. Supplemental table containing the full data set used in this work including subject age, body mass index (BMI), diagnostic class (i.e. ME/CFS or control), cohort reference and illness duration as well as the normalized and coded values for each cytokine species measured in each subject. **Table S7.** Supplemental table containing symptom severity based on the Multidimensional Fatigue Inventory (MFI) for all ME/CFS subjects aged 18 years and older. (XLS 86 kb) Additional file 4: Figure S2. Structure of a simple prototype classification model for ME/CFS. A diagrammatic representation of the step-by-step use of an initial linear model for the classification of ME/CFS versus age and BMI-matched healthy control subjects adjusted for duration of illness. (PDF 128 kb)
