# Supplementary figures and images for: Illness progression in chronic fatigue syndrome: a shifting immune baseline
Source: BMC Immunol. 2016 Mar 10;17:3. doi: 10.1186/s12865-016-0142-3 (PMC4785654; doi:10.1186/s12865-016-0142-3)

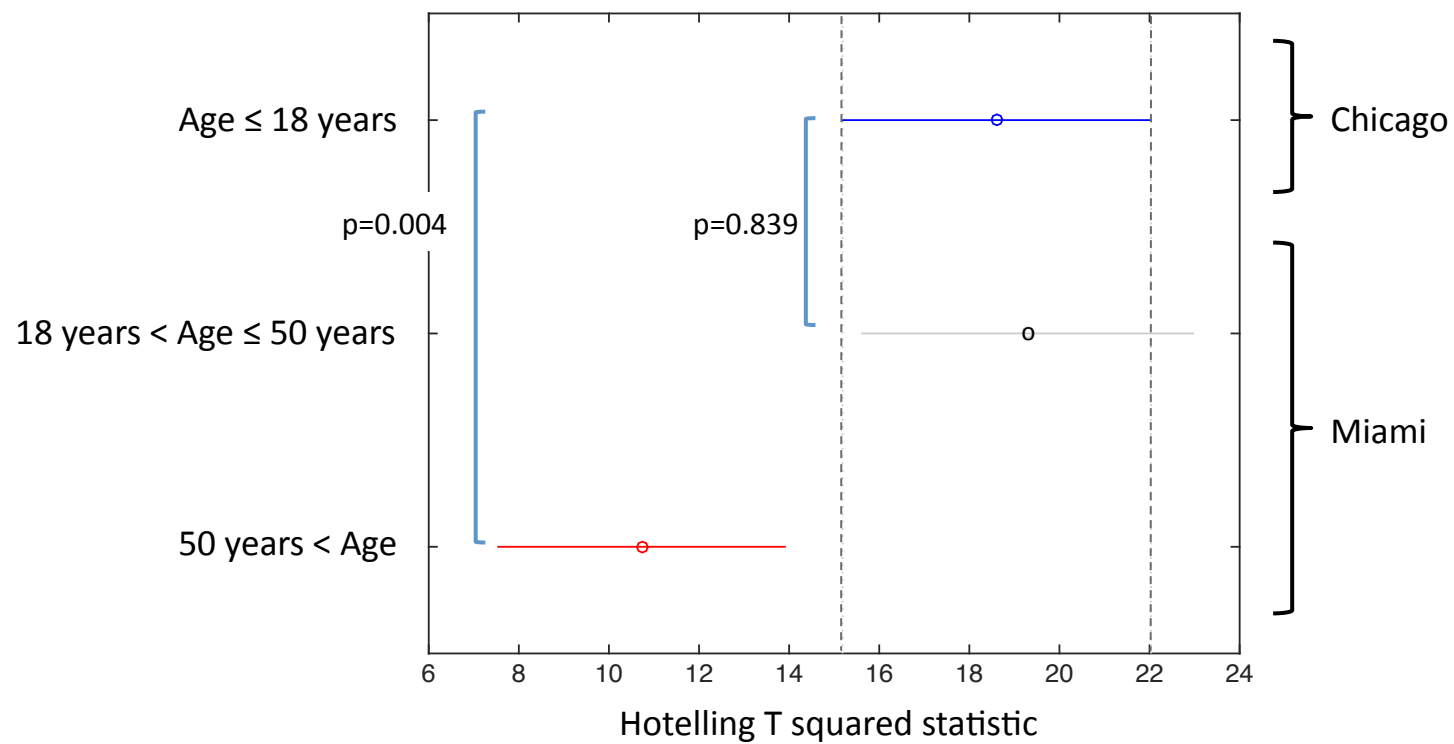

Figure S1

Supplement: Additional file 2: Figure S1. — Site-to-site variability in samples from healthy control subjects. Hotelling’s T squared residual distance separating samples collected in all 3 groups of healthy control subjects from a principal component analysis (PCA) model describing the co-expression patterns linking 16 cytokines. Middle-aged and adolescent groups were statistically comparable (p = 0.839) despite samples being collected at separate sites. (PDF 37 kb) [file 12865_2016_142_MOESM2_ESM.pdf]

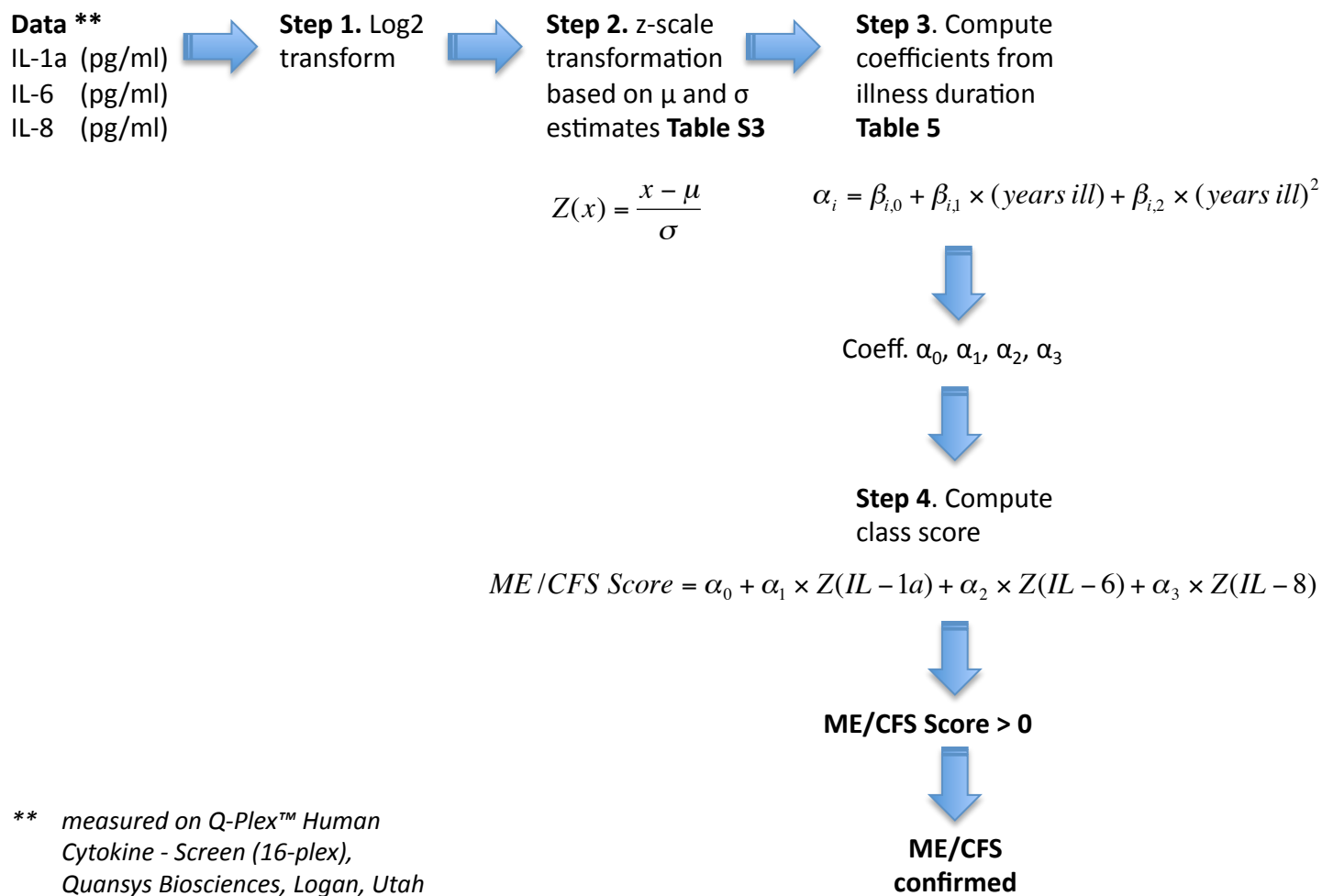

Figure S2

Supplement: Additional file 4: Figure S2. — Structure of a simple prototype classification model for ME/CFS. A diagrammatic representation of the step-by-step use of an initial linear model for the classification of ME/CFS versus age and BMI-matched healthy control subjects adjusted for duration of illness. (PDF 128 kb) [file 12865_2016_142_MOESM4_ESM.pdf]
